# Co-infections as Modulators of Disease Outcome: Minor Players or Major Players?

**DOI:** 10.3389/fmicb.2021.664386

**Published:** 2021-07-06

**Authors:** Priti Devi, Azka Khan, Partha Chattopadhyay, Priyanka Mehta, Shweta Sahni, Sachin Sharma, Rajesh Pandey

**Affiliations:** ^1^INtegrative GENomics of HOst-PathogEn Laboratory, CSIR-Institute of Genomics and Integrative Biology, New Delhi, India; ^2^Academy of Scientific and Innovative Research (AcSIR), Ghaziabad, India

**Keywords:** co-infection, HIV, HCV, MTB, oxidative stress, immune response, disease severity

## Abstract

Human host and pathogen interaction is dynamic in nature and often modulated by co-pathogens with a functional role in delineating the physiological outcome of infection. Co-infection may present either as a pre-existing pathogen which is accentuated by the introduction of a new pathogen or may appear in the form of new infection acquired secondarily due to a compromised immune system. Using diverse examples of co-infecting pathogens such as Human Immunodeficiency Virus, *Mycobacterium tuberculosis* and Hepatitis C Virus, we have highlighted the role of co-infections in modulating disease severity and clinical outcome. This interaction happens at multiple hierarchies, which are inclusive of stress and immunological responses and together modulate the disease severity. Already published literature provides much evidence in favor of the occurrence of co-infections during SARS-CoV-2 infection, which eventually impacts the Coronavirus disease-19 outcome. The availability of biological models like 3D organoids, mice, cell lines and mathematical models provide us with an opportunity to understand the role and mechanism of specific co-infections. Exploration of multi-omics-based interactions across co-infecting pathogens may provide deeper insights into their role in disease modulation.

## Introduction

Typically, an infectious disease is thought to be caused by a single pathogen. However, concomitant infections by two or more pathogens are often observed in the real-world context. Co-infections may be detrimental, insignificant or beneficial for the disease outcomes depending on the levels of interactions, such as modulation of host response, diagnostic and therapeutic interventions (McArdle et al., 2018). Broadly, the interactions may be positive or negative. Positive interaction includes when pathogens in the host system act synergistically and lead to deterioration of disease symptoms, severity and outcomes. Negative interaction occurs when one pathogen impedes the growth of another pathogen through competition, parasitism or interference (Hoffman et al., 2006). When multiple pathogens co-infect a host, one pathogen may influence the replication and disease severity caused by the other. This is mostly observed during viral infections and is known as viral interference. Viral interference may lead to early clearance of one infection and persistence of the other one (Kumar et al., 2018). This is regulated by interferons (IFNs), transacting protease, defective interfering particles, and non-specific double-stranded RNA (Salas-Benito and De Nova-Ocampo, 2015; Kumar et al., 2016). Apart from the direct interactions between co-infecting pathogens, host response also plays a pivotal role in shaping the outcome of co-infections.

**FIGURE 1:**
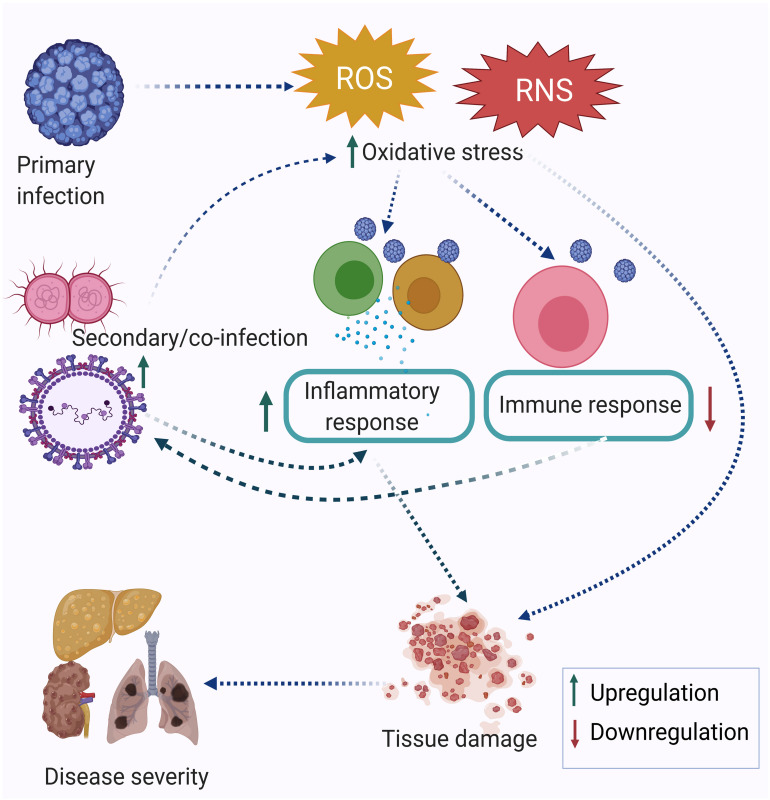
Co-infection modulating oxidative stress, immune response, and disease severity. The primary infection leads to an increase in oxidative stress, which is further enhanced by the secondary infection and eventually leads to dysregulation of the immune response. Taken together, this leads to tissue damage and acute disease outcomes.

Research performed in the last two decades has established that infection triggers oxidative stress by inducing the production of reactive oxygen species (ROS) and reactive nitrogen species (RNS). Oxidative stress-mediated epithelial barrier dysfunction and lung tissue injury have also been reported in acute respiratory infections. As a result of the oxidative burst during acute respiratory infection, the chances of secondary infection get increased (Ivanov et al., 2017). In many cases, oxidative stress is more pronounced during co-infection compared to a single infection alone (Rajopadhye et al., 2017). The memory T cells generated after one infection may alter the degree of immune response elicited against subsequent infection or co-infection. This is known as the heterologous immune response. Many immune cells are involved in a heterologous immune response, which may induce either protective or immune-pathological reaction upon a secondary infection or co-infection. The extent of oxidative stress and immune response together regulate the disease severity. Figure 1 depicts the inter-relationship between oxidative stress, inflammatory response, immune response and disease severity during co-infection by multiple pathogens. Co-infections such as in the case of *Mycobacterium tuberculosis* (MTb) or Hepatitis C virus (HCV) infection with Human Immunodeficiency Virus (HIV) are synergistically associated and have multi-faceted detrimental effects on the host. Apart from the cellular effect of increased oxidative stress, studies have reported the regulation of one infection by another co-infecting pathogen. For example, *Mycobacterium* infection exacerbates HIV-1 infection by oxidative stress induction mediated through the exosomes released from the infected macrophages (Tyagi et al., 2020). This review attempts to highlight the often ignored aspect of co-infections in infectious disease biology, albeit with limited specific examples, including its relevance in the context of the current Coronavirus disease-19 (COVID-19) pandemic. Finally, studying co-infections enables a better understanding of the effectors and outcomes of co-infections in terms of the role of oxidative stress and immune response.

### Oxidative Stress Response

Reactive oxygen species are highly reactive oxygen ions due to the presence of unpaired electrons in their outermost shell. Examples of ROS include superoxide (O_2_^•−^), hydroxyl radical (OH•), nitric oxide (NO•), hydrogen peroxide (H_2_O_2_), singlet oxygen (^1^O_2_), and ozone/trioxygen (O_3_). Mitochondria and peroxisomes are the major sites for production of ROS (Liou and Storz, 2010). It plays many important roles in physiological processes, including signaling, apoptosis and maintenance of cellular homeostasis. ROS is scavenged by antioxidant enzymes such as catalase, superoxide dismutase (SOD), and glutathione (GSH). An imbalance between the production and scavenging of ROS disrupts the redox homeostasis, leading to a state of oxidative stress with many detrimental effects on the cells. Elevated levels of ROS have been reported in many diseases, including infections (Bolukbas et al., 2005; Ivanov et al., 2017).

The immunological consequences of HIV infection open the door for opportunistic co-infecting pathogens to invade. One such co-infecting pathogen in HIV patients is MTb. Awodele and group reported reduced levels of antioxidant enzymes (GSH, SOD, and catalase) in HIV–MTb co-infected patients compared with patients having HIV infection only. The same group also reported an elevated level of malondialdehyde (MDA), a marker of lipid peroxidation, in HIV–MTb co-infection compared to HIV infection only (Awodele et al., 2011). MTb infection induces both mononuclear and polynuclear phagocytes to produce the ROS. Increased levels of ROS promote tissue injury and inflammatory response, which in turn, leads to immune-suppression (Jack et al., 1994; Rajopadhye et al., 2017). The situation gets further complicated in people with compromised immune systems, like patients with HIV infection, exacerbating the disease complications and possible morbidity. Reduced levels of antioxidant markers such as Vitamin A, C, E, selenium, GSH, SOD, catalase, as well as an increased level of MDA are observed in MTb patients co-infected with HIV (Rajopadhye et al., 2017; Gravier-Hernández and Gil-del Valle, 2018).

**FIGURE 2:**
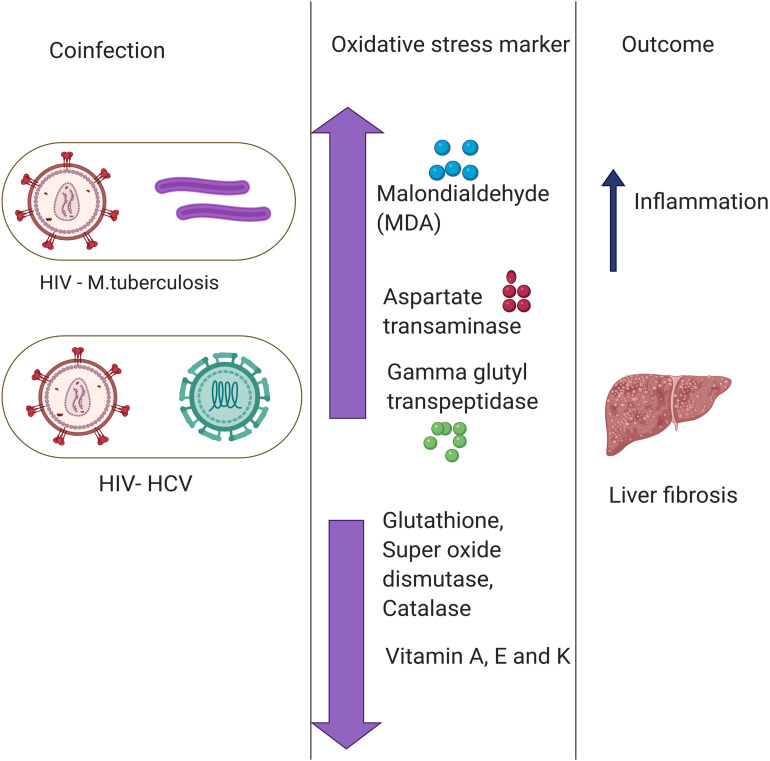
Role of co-infections in regulating oxidative stress. Co-infection of Hepatitis C Virus (HIV) with *Mycobacterium tuberculosis* (MTb) and human immunodeficiency virus (HCV) primes to a disproportion of oxidative stress and antioxidant markers leading to various disease outcomes such as liver fibrosis and inflammation. There are multiple Oxidative stress markers as highlighted above.

Another common example of co-infection is the concurrent infection with HIV and HCV. Multiple studies have reported a significant difference in the levels of oxidative stress markers and antioxidant stress markers between individuals with HIV or HCV co-infection and those with only one infection (Stehbens, 2004; Grønbaek et al., 2005; Baum et al., 2011). The chronic HIV infection leads to chronic oxidative stress with severe liver damage, cirrhosis, fibrosis, and carcinogenesis (De Maria et al., 1996). HCV infection leads to depleted levels of GSH, which causes immune suppression (Müller et al., 2017). The immune suppression, in turn, deteriorates the health condition of the patient in case of co-infection with HIV. Increased oxidative stress together with a compromised antioxidant defense system is one of the major causes of severe liver damage in HIV–HCV co-infection. Malondialdehyde, a marker of oxidative stress, was found to be elevated in patients with HCV or HIV infections (Paradis et al., 1997; Parola and Robino, 2001). Besides, antioxidant markers such as Vitamin A and E, zinc and selenium, are also found to be depleted in HIV patients. The combined effect results in a higher prevalence of liver fibrosis (Parola and Robino, 2001). Furthermore, in an *in vitro* study, it was found that treatment with antioxidants reduces the oxidative stress and disease severity induced by HIV and HCV (Price et al., 2006). A lower mitochondrial DNA copy number was observed in the HIV–HCV co-infected patients compared to either HIV or HCV infection alone, and this causes a further increase in oxidative stress (de Mendoza et al., 2005). It is presumed that the enhanced oxidative stress found in HIV–HCV co-infection may contribute to more rapid progression of liver fibrosis by stimulating HCV replication and enhanced production of ROS in hepatocytes (Farinati et al., 1995; Paradis et al., 1997; García-Mediavilla et al., 2005). On the flipside, multiple studies have evidenced ROS mediated modulation of viral replication. ROS was found to promote the HIV replication (Pace and Leaf, 1995; Ranjit et al., 2018). However, the effect of elevated ROS on HCV replication is yet to be fully understood (Medvedev et al., 2016; Anticoli et al., 2019). These pieces of evidence highlight an important role of co-infection in modulating the oxidative stress response and shaping the disease outcomes (Figure 2).

### Immune Responses

Within the ambit of this review, this section focuses on the immunological aspects of HIV–MTb, and HIV–HCV co-infection. We have also attempted to give an insight into HIV and MTb co-infections with SARS-CoV-2, even though research involving SARS-CoV-2 co-infections is a work in progress. Besides the already mentioned role of oxidative stress in mediating immune suppression, the former is also known to induce Unfolded Protein Response (UPR), which in turn stimulates the immune response (Grootjans et al., 2016). The oxidative stress-mediated regulation of immune response, therefore, depends on the extent of oxidative stress during the course of infection.

**FIGURE 3:**
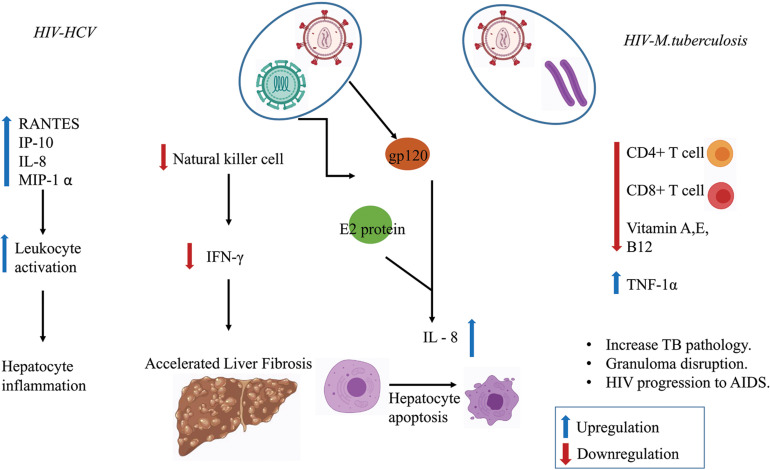
Co-infection in regulating the immune response. Various chemokines and immune cells show dysregulation during co-infection state which enhances disease severity such as liver fibrosis, hepatocyte inflammation, increase in MTb pathology, Granuloma disruption, and the progression of HIV to Acquired Immunodeficiency Syndrome (AIDS).

Several *in vitro* studies have shown that MTb infection stimulates the release of interleukin (IL-6) and the production of C-reactive protein (CRP)–a marker of inflammation (Bekker et al., 1998; Unsal et al., 2005; Rath et al., 2013). CRP level was found to be more pronounced in the HIV–MTb co-infection group compared to MTb infection alone (Rajopadhye et al., 2017). HIV–MTb co-infection also enhances the levels of pro-inflammatory cytokines such as Tumor Necrosis Factor-alpha (TNF-α) that further increases the replication of the virus and promotes the progression of the HIV to Acquired Immunodeficiency Syndrome (AIDS) (Kitaura et al., 2001). Figure 3 summaries the immunological outcomes of HIV–MTb and HIV–HCV co-infections.

Several studies have demonstrated the cellular intricacies of the dysregulated immune system in infection and co-infections. While CD4+ T cell depletion is one of the major hallmarks of HIV infection, infection with HIV also results in the depletion of *M. tuberculosis*-specific CD4+ and CD8+ T cells. This results in a marked decrease in IL-2, IL-4, IL-5, and IL-13 levels in both latent and pulmonary MTb infection, thereby promoting disease progression (Ahmed et al., 2016; Amelio et al., 2019). Moreover, it is known that HIV is a main driver in the re-emergence of primary and reactivation of TB. Reactivation risk of latent TB is increased in patients after acquiring HIV. Interestingly, patients with HIV are more liable to TB, irrespective of CD4+ T cell levels but at the same time, risk increases with decrease in CD4+ T cell levels (Lin et al., 2012). Amelio et al. (2019) reported that serum levels of IL-1 (IL-6, IL-23, IP-10, and CRP in HIV–MTb co-infected patients are reduced compared to MTb infection alone. CD4+ T depletion in immune-compromised HIV infected patients promote the infection of other opportunistic pathogens such as *Candida albicans* and cytomegalovirus (CMV). Ample of evidences exhibit that HIV infected patients are more susceptible to mucosal candidiasis during early stage but CMV pathogenesis is evident during more severe reduction of CD4+ T cell in late phase of HIV infection (Anglaret et al., 2012).

In HIV infected patients, viral Tat protein stimulates the production of ROS, which may then activate the nuclear factor κB (NF-κB) and increase the viral transcription (Schreck et al., 1991; Sacktor et al., 2004). At the same time, many *in vitro* and *in vivo* studies have reported that MTb infection enhances the replication of HIV (Goletti et al., 1998; Hoshino et al., 2007, 2002). However, another study reported inhibition of HIV-1 replication by the MTb infection-induced IL-10 (Goletti et al., 1998; Souriant et al., 2019). In HIV infected patients, restoration or improvement of immune responses is mediated by antiretroviral therapy (ART). ART increases the life expectancy and decreases the mortality rate in HIV infected persons. But initiation of ART exaggerates immune response toward many pathogens that cause the immune reconstitution inflammatory syndrome (IRIS). Innate and adaptive immune system factors such as CD4+ T-cells, CD8+ T-cells, natural killer cells, macrophages, complement system, Toll -like receptors and pro-inflammatory cytokines promote the pathogenesis of IRIS. Risk factors contributing toward development of IRIS are high antigen load, low CD4+ cell counts, and scarcity of time between the start of ART and treatment for opportunistic pathogens. TB is considered among the most frequently reported pathogens linked with IRIS and studies have shown the occurrence of TB-IRIS within 2 months of ART initiation (Nelson et al., 2017; Aggarwal et al., 2018). Most common clinical manifestations of TB-IRIS are fever, lymphadenopathy and severe respiratory cues. Disseminated tuberculosis with associated acute renal failure, tuberculous meningitis, skin or visceral abscesses, osteomyelitis, epididymo-orchitis, abdominal TB-IRIS with non-specific abdominal pain, bowel perforation, and obstructive jaundice are also possible (Cevaal et al., 2019; Stek et al., 2020).

On similar lines, many studies have reported a higher extent of the immune-dysregulation in HIV–HCV co-infection compared with HIV or HCV infection alone. Interferons (IFN) are the key to inducing an antiviral immune response. Flynn et al. (2012) reported a significantly lower IFN-γ response, both in terms of magnitude and breadth, in HIV patients co-infected with HCV, when compared to HCV infection alone. This detrimental effect on IFN-γ in acute HCV infected patients co-infected with HIV correlates with the peripheral CD4+ T cell count. IP-10, an interferon-induced chemokine, is upregulated during HIV–HCV co-infection in comparison to HCV or HIV infection alone and the IP-10 level is correlated with the extent of hepatic inflammation and liver damage (Roe et al., 2007). In HepG2 cells co-infected with HCV and HIV, the HCV envelope protein (HCV-E2) and HIV envelope protein (HIV-gp120) together trigger the p38 mitogen-activated kinase (MAPK) pathway. This stimulates IL-8, a potent chemo-attractant and inducer of diseases such as rheumatoid arthritis, respiratory distress and sepsis. The IL-8 stimulation was found to be higher in the case of HIV–HCV co-infection than in cases of mono-infection (Kunkel et al., 1994; Balasubramanian et al., 2003). Apart from the immune-dysregulation, HIV–HCV co-infection is also linked to poor CD4+ T cell restoration in patients undergoing antiretroviral therapy (ART) (Shmagel and Saidakova, 2018). Taking together, these pieces of evidence of dysregulated immune and inflammatory responses as well as poor response to therapy may partly explain the reason for the rapid development of liver cirrhosis and hepatocarcinoma in HCV patients co-infected with HIV.

Many evidence toward co-infections with SARS-CoV-2 have been discovered during the current COVID-19 pandemic. Among the co-infecting pathogens, rhinovirus, RSV, *Chlamydia pneumoniae*, IAV, H1N1, *Staphylococcus aureus*, *Pseudomonas aeruginosa*, *Staphylococcus pneumoniae*, and *Klebsiella pneumoniae* are commonly observed in SARS-CoV-2 positive patients (Chen et al., 2020; Kim D. et al., 2020; Ma et al., 2020; Zhu et al., 2020; Burrel et al., 2021). Several cases of co-infection of MTb and HIV with SARS-CoV-2 have been reported as well, but there is a lack of any clear evidence supporting the role of co-infection in modulating the disease severity (Kanwugu and Adadi, 2020; Stochino et al., 2020). SARS-CoV-2 and *M. tuberculosis* may interact synergistically and affect the pathology of infection within the host, considering the similar route of cell entry, immune response and immune evasion (Tapela et al., 2020). SARS-CoV-2 and MTb, both infect the same type of cells, which include type II pneumocytes in the lungs and macrophages (Qian et al., 2013; Ganbat et al., 2016; Abassi et al., 2020). A case study by Khayat et al. (2021) reported progression of latent MTb infection (LMTBI) to an active MTb infection following a marked CD4+ T cell depletion associated with COVID-19. Another study involving COVID-19 reported a lower immune response (as measured by IFN-γ response against MTb antigen and SARS-CoV-2 S protein antigen) in MTb-COVID-19 and LMTBI-COVID-19 patients when compared to COVID-19 patients (Petrone et al., 2021). The aforementioned pieces of evidence indicate that depletion of T lymphocytes in COVID-19 patients may be a matter of concern; especially for people with LMTBI. Also, the lower levels of SARS-CoV-2 specific immune response in COVID-19 patients co-infected with MTb may increase the chance of re-infection with SARS-CoV-2.

Cases of HIV co-infection with SARS-CoV-2 have also been reported, wherein SARS-CoV-2 is found to accelerate the T lymphocyte exhaustion in HIV patients. The T lymphocyte depletion was more pronounced in HIV-SARS-CoV-2 co-infected patients without ART. IL-10 and tumor growth factor β (TGFβ) levels were also found to be elevated in patients co-infected with HIV and SARS-CoV-2, but much lower in patients undergoing ART (Sharov, 2021). Hyper-immune activation and resulting cytokine storm is believed to be the key player in COVID-19 disease severity and mortality (Hojyo et al., 2020). However, a case study by Suwanwongse and Shabarek (2020) reported a much higher mortality rate (7 out of 9 patients) in HIV-COVID-19 co-infected patients with a significantly lower CD4+ T cell count, than among COVID-19 patients without HIV. This is in contrast to the idea that the immune suppression by HIV infection may reduce the severe immunological consequences of COVID-19. Also, other studies, including another study by the same group recorded a lower mortality rate among HIV–COVID-19 patients compared to previous studies (Altuntas Aydin et al., 2020; Blanco et al., 2020; Marimuthu et al., 2021; Suwanwongse and Shabarek, 2021). One case study by Wang et al. reported a longer disease course and slow production of specific antibodies, when simultaneously infected by SARS-Cov-2 and HIV, may severely damage the immune system (Wang et al., 2020). The same group suggested that depletion of CD4+ lymphocytes occur and the remaining T-cells may have abnormal response to antigens during such co-infection. Interestingly, it is evident in severe patients contracted with COVID-19 that CD4+ T and CD8+ T levels were low (Zhang and Wu, 2020). Such retrospective study leads to a understanding that irrespective of HIV status, patients with COVID-19 still have low counts of CD4+ T cells. However, some studies reported poorer COVID-19 related outcomes in patients living with HIV (Ceballos et al., 2021; Tesoriero et al., 2021). The divergent findings in the case of HIV–SARS-CoV-2 co-infection warrant a thorough investigation into the immunological aspects of SARS-CoV-2 co-infection with HIV.

### Disease Severity

As discussed in the previous sections, increased oxidative stress and dysregulated immune function are evident during co-infections. Co-infections by multiple pathogens may play an important role in ameliorating or deteriorating disease outcomes. Opportunistic infection of MTb in HIV patients leads to a higher rate of mortality. The main reason for the compromised immune system in HIV patients is the progressive loss of CD4+ T cells in the lymphoid tissues, blood and mucosa, which significantly contributes to the increased risk of developing active MTb (Bruchfeld et al., 2015). Individuals having latent MTb possess a high propensity for reactivation of MTb if co-infected with HIV (Diedrich and Flynn, 2011). Both HIV and MTb act synergistically and in the process cripple the various arms of the host immune system, leading to a subsequent increase in mortality in the absence of any recommended standard of care (Whalen et al., 1995; Modjarrad and Vermund, 2010). Evidence suggests that HIV–MTb co-infection enhance/synergize each other in individuals and lead to immune dysfunction causing premature death, if left untreated. According to the World Health Organization (WHO) reports, worldwide, an estimated 10 million people were infected with MTb in 2019, among those nearly 8.2% included those living with HIV (Tuberculosis data, 2020). Another WHO report says one-third of the HIV infected patients have MTb and have up to 50 times higher risk of MTb infection compared to HIV negative individuals [Fact Sheet on Tuberculosis (TB), WHO]. An estimated 90% death rate was observed in HIV patients within 1 month of contracting MTb, if not treated properly.

Globally, an estimated 130 million individuals are infected with chronic hepatitis C virus (HCV). Among these, 4–5 million persons are co-infected with HIV (Operskalski and Kovacs, 2011). Infection with HIV enhances the HCV related disease severity such as cirrhosis, fibrosis, and end-stage liver disease (ESLD). Co-infected individuals are three times more prone to develop cirrhosis as compared to mono-infected individuals (Operskalski and Kovacs, 2011). A 20-years prospective study showed that co-infected drug users (DUs) receiving highly active antiretroviral therapy (HAART) had a higher risk of liver-related death compared to HCV mono-infected DUs (Smit et al., 2008). The accelerated liver disease in co-infected individuals is proposed to be due to the dysregulated immune system, increased oxidative stress, direct viral effects and decline of HCV specific T cells responses (Roe and Hall, 2008; Kim and Chung, 2009; Rotman and Liang, 2009). HIV infection is known to dysregulate the levels of cytokines like IL-4, IL-5, and IL-13, which in turn, accelerates liver fibrosis and inflammation (Roe and Hall, 2008). Further exacerbation of liver fibrosis is caused due to the imbalance of extracellular matrix (ECM) characterized by accumulation of ECM and declined deterioration of connective tissue proteins. A large family of zinc-dependent endopeptidases, matrix metalloproteinases (MMPs) control the degradation of ECM. Altered level of MMPs production could cause inflammation, wounds, invasion of cancer cells and liver fibrosis (Friedman, 2008a, b; Zhao et al., 2013). Study revealed that altered level of MMP and tissue inhibitors of metalloproteinases (TIMP) expression are linked to hepatic fibroproliferation in chronic Hepatitis C. HIV/HCV co-infected patients with more advance reduction of CD4+ T cell display enhanced circulating TIMP-1 level and thus promote liver fibrosis (Mastroianni et al., 2014). It is well known fact that low level of hyaluronic acid (HA) is associated with the low risk of liver disease progression. Toward predicting the liver disease progression in HIV/HCV co-infection, detecting the HA plasma detection alone or in combination with other methods, may be a beneficial way of monitoring (Peters et al., 2013). Apoptosis of hepatocytes through a Fas/FasL pathway has been found to be enhanced in HIV–HCV co-infection that results in hastening the liver disease (Kim and Chung, 2009). The accumulation of CD8+ T cells in the liver of co-infected individuals enhances the production of TNF-α and other inflammatory mediators (Kuntzen et al., 2008; Nakamoto et al., 2008; Vali et al., 2008). This further exacerbates the tissue damage and liver fibrosis when compared to HCV infected individuals. Replication of HIV in the hepatocytes and hepatic stellate cells (HSC) stimulates the production of collagen and pro-inflammatory cytokines that further promote the fibrosis (Blackard and Sherman, 2008; Tuyama et al., 2010). HIV–HCV co-infection has been reported to enhance the severity of kidney-related diseases such as acute renal failure and proteinuria when compared to the HIV infection alone (Wyatt et al., 2008). Also, HIV–HCV co-infection related nephropathies increased the mortality rate compared to the HCV mono-infection (Izzedine et al., 2009). Cardiovascular diseases are also found to be more prevalent in the HIV–HCV co-infection when compared to the HIV mono-infection. Co-infection with HCV enhances the chances of acute myocardial infarction in the HIV infected individuals (Bedimo et al., 2010). HIV–HCV co-infected individuals treated with HAART showed higher endothelial dysfunction due to elevated levels of circulating soluble cellular adhesion molecules (CAM_*S*_) released by the vascular endothelium, soluble intercellular adhesion molecule-1 (sICAM-1) and vascular adhesion molecule-1 (sVCAM-1) (de Castro et al., 2010).

In contrast to the above-mentioned cases, wherein co-infection deteriorates the disease outcome, co-infection of Influenza A viruses and rhinoviruses, both of which invade the respiratory tract, is found to be associated with reduced disease severity (Greer et al., 2009; Esper et al., 2011; Jain et al., 2015; Nolan et al., 2018). A mouse model-based study by Gonzalez et al. found that prior inoculation of a rhinovirus strain 1B (RV1B) suppressed the severity of influenza infection in a time and dose-dependent manner. The decrease in mortality and disease severity was not associated with a reduction in the viral titer or replication, but prior inoculation with rhinoviruses induced early inflammatory response in the lungs that helped in clearance of influenza virus strain (Gonzalez et al., 2018). Another study by Shinjoh et al. (2000) reported that IAV infected cells when co-infected with RSV had reduced IAV titers, without altering the direct replication of IAV after 12 h post-infection. Ample evidence exhibit role of IAV facilitating the infection of *S. pneumoniae* in respiratory tract. Type I interferon induced by IAV infection sensitizes the host for other bacterial co-infections like *S. pneumoniae* and Mtb and often lead to serious consequences (Shahangian et al., 2009; Redford et al., 2014). Although there is no direct involvement of IAV induced IFN in regulating the SARS-CoV-2 co-infection, studies suggested that the higher infectivity of SARS-CoV-2 in IAV infected patients may be due to ACE2 expression, which is IFN-stimulated gene (Ziegler et al., 2020; Bai et al., 2021). Virus promotes the bacterial super-infection by enhancing the availability of receptors. Cryptic receptors for bacterial adherence are facilitated by the influenza virus neuraminidase that cleaves sialic acid and disrupt sialylated mucins that can function as decoy receptors for the bacteria (McCullers and Bartmess, 2003; McCullers, 2004). In addition to dysregulated immune responses and imbalance of ROS, excess of metal such as iron may contribute toward the severity and outcomes of infections. Study revealed that excess amounts of iron promote the viral replication and multiplication of mycobacteria (Lounis et al., 2001; Chao et al., 2019). The proteins involved in virulence and oxidative stress response in MTb are regulated by the iron dependent regulator, IdeR (Kurthkoti et al., 2015). It is reasonable to anticipate that the excess iron may be the cause of more severe outcome during co-infection as excess iron has potential to impact infectivity of different pathogens independently. High serum iron was detected in HIV patients, which, might contribute to further CD4+ T cell depletion (Banjoko et al., 2012; Chang et al., 2015). Elevated hepatic iron level was also reported in HCV infected patients (Maier, 1997; Price and Kowdley, 2009). The elevated iron level was reported to elevate 8-hydroxy-2’-deoxyguanosine (8-OHdG) level, resulting in hepatic oxidative stress and DNA damage, leading to liver fibrosis (Fujita et al., 2008). Iron chelating agents such as silybin and DFO may be a better therapeutic for preventing pathogen multiplication (Agoro and Mura, 2019). The varying outcomes of co-infections necessitate further research into the dynamics of interactions between co-infecting pathogens to enable better management and treatment of infectious diseases.

### Experimental Models to Study Co-infection

**FIGURE 4:**
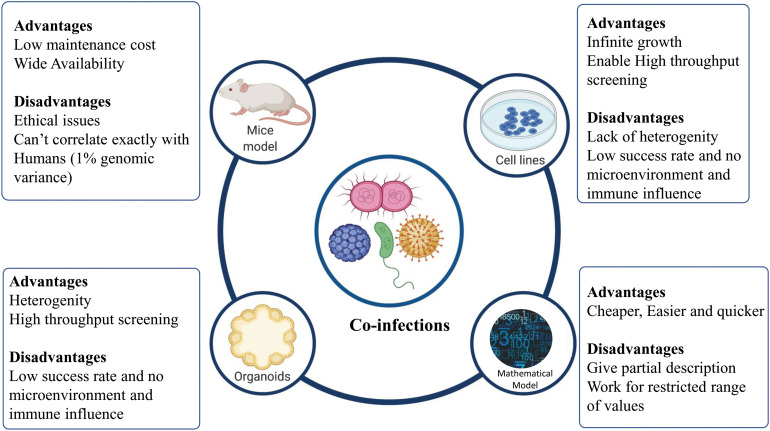
Different models to study co-infection. Various model systems (mice models, cell lines, organoids, and mathematical models) can be potentially harnessed to decipher the role of co-infections. The figure also shows the advantages and disadvantages associated with various model systems mentioned.

To understand the drivers of disease severity, clinical implications, diagnosis, and therapeutic management in case of infectious diseases, experimental model systems are required to recapitulate and mimic the patho-physiology of the host-pathogen interactions. Different model systems, as depicted in Figure 4, can be potentially harnessed to decipher the role of co-infections in modulating the disease outcomes.

Cell lines provide the most rudimentary information about the nature of a host-pathogen interaction, if cells are permissive to their division/replication. For example, the examination of HIV-HBV interactions within hepatic cell lines has been demonstrated by Iser et al. (2010). However, cell lines must be maintained specially to balance selective pressure for growing pathogens. For a more detailed insight into mechanistic details of the infection, a “complex architect of tissue” is the prerequisite. Different animal models are also available, which allow the study of co-infections. Animal models have been used to mimic different diseases caused by co-infecting viruses, bacteria, and virus-bacteria. For example, co-infection of pulmonary tuberculosis with HIV-1 was studied in humanized mice model by Rebecca et al. (Nusbaum et al., 2016). Using animal models allows deciphering the patho-physiology of a particular co-infection for better understanding and therapeutic management of the disease. However, mimicking human diseases in animal hosts presents significant challenges owing to the host-dependent restriction mechanisms of most pathogens (Bakaletz, 2004). An *in vitro* 3D Organoid model provides a possible means to bridge the gap between the 2D and the *in vivo* model, to understand the modulators of host-pathogen interaction in deciphering the pathogenesis, defining the species barrier and identifying cellular niches for bacteria or viruses (Rossi et al., 2018; Kim J. et al., 2020).

Organoids are 3D structures, developed from multipotent tissue adult stem cells or most often from induced pluripotent cells. Organoids can be used experimentally to test the new investigational therapeutics against the co-infecting pathogens. However, a lack of the dynamic microenvironment mimicking the human body, blood vessels, nerves and immune influence (inter-organ communication) can be counted as one of the limitations of organoid models of infection (Kim J. et al., 2020). Apart from these experimental model systems, mathematical models have been developed to better understand the disease dynamics and to devise better therapeutic regimens (Esteva and Vargas, 2003; Vickerman et al., 2012; Corson et al., 2013). Different co-infecting pathogens such as RSV, rhinovirus, IAV, Human para-influenza virus, and human metapneumovirus infection dynamics can be studied via a mathematical model devised by Pinky and Dobrovolny (2017). Other than understanding the mechanistic details of co-infection biology, such models can be employed to simulate and understand the disease pathophysiology, decipher host-pathogen interactions and for the screening of drugs and other therapeutic interventions against co-infecting pathogens.

## Discussion

**FIGURE 5:**
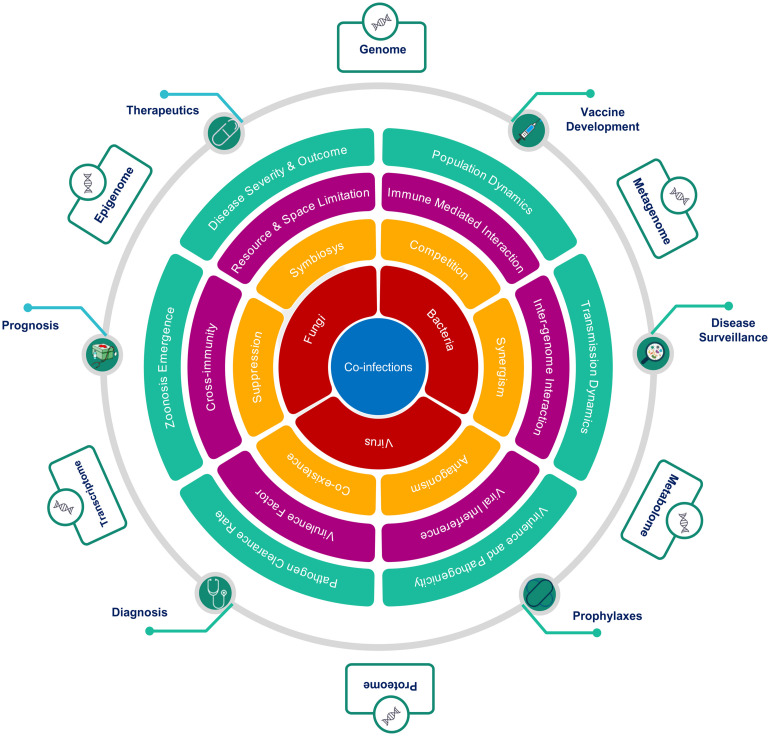
Hierarchical role of co-infections as cause, modulator and effector of clinical outcomes. The figure highlights the importance of an integrative approach across multiple hierarchies to study co-infections, from the perspective of, **(A)** interacting microorganisms (Red), **(B)** nature of interactions between them (Yellow), **(C)** mechanisms mediating interactions (Magenta), **(D)** macro level effects of co-infections (Green), **(E)** Public health interventions affected by co-infections (Green circles), and (F) multi-omics approaches to study of co-infections (Outer boxes).

Co-infection biology is known to be modulated by the nature of interaction at multiple hierarchies between interacting microorganisms within the common host, which explains the complex non-linear dynamics of infectious diseases severity and clinical outcome (Figure 5). The degree of facilitation or inhibition between interacting pathogens, dependent upon the co-infecting species, in turn, decides the severity of a disease. A mechanistic understanding of the nature of these interactions, whether synergistic or antagonistic, has major consequences for treatment-specific decision making. The interactions between microbes at the cellular level are driven by a multiplicity of factors. Changes in cell surface receptor presentation may be altered by a pathogen, which may, in turn, lead to super-infection suppression or exclusion. Co-infecting pathogen dynamics within the host are driven by ecological constraints inclusive of resource and space limitation. Co-infecting microbes modulate the infection outcomes by altering the nature and extent of immunological response within the host. A classical case in point is the HIV infection, which compromises the host immune system thereby, making the host more susceptible to co-infection by other opportunistic pathogens. At the host level, inter-pathogen interactions may lead to altered disease epidemiology affecting the mortality and morbidity associated with an infectious disease (Pandey et al., 2021; Figure 5). Co-infection increases the possibility of genetic recombination and reassortment leading to the emergence of novel antigenic variants thus affecting the efficacy of drugs, treatment regimen and vaccines. In the rarest of instances, this may even lead to the evolution of new zoonotic infections. Co-infection biology is also important to evaluate the nature of response to pharmacological interventions. Patients with co-infections, when subjected to antimicrobial chemotherapies, may facilitate the emergence of multi-drug resistant species and propagation of Antimicrobial Resistance (AMR). Population-level consequences of co-infections are, therefore, important to consider for designing, optimizing, and delivering Public Health Interventions in diagnostics, therapeutics, prophylaxis and prognosis of infectious diseases. The non-linear disease outcomes in the case of infectious diseases are important considerations for disease surveillance and public health policymaking at the societal level.

To conclude, infectious disease dynamics has an important component of co-infections or co-presence of microbes (potential pathogens). The degree and nature of this conditioning and the underlying mechanisms remain largely unexplored although its potential has been highlighted by the examples discussed above. Multi-Omics approaches, as depicted in Figure 5, may provide suitable tools and techniques to enable a clearer understanding and elucidation into the role and significance of co-infections in modulating infectious disease outcomes. The co-infection outcome can be infested at multiple hierarchies inclusive of genomic, transcriptomic, epigenomic, metagenomic, proteomic, and metabolomic. This will have functional impact on disease surveillance, diagnosis, detection, treatment regimen, vaccine development and efficacy. Thus, the dynamics of primary infection, secondary infection, pre-existing infection and opportunistic infection in time and space within population/s have important role toward disease trajectory and outcome.

## Author Contributions

PD, AK, PC, PM, SSa, SSh, and RP wrote the manuscript. RP coordinated the study and funding for the work. All authors contributed to the article and approved the submitted version.

## Conflict of Interest

The authors declare that the research was conducted in the absence of any commercial or financial relationships that could be construed as a potential conflict of interest.
